# Annual Review of Systemic Medical Treatment for Colorectal Cancer in 2025

**DOI:** 10.1002/cai2.70057

**Published:** 2026-04-10

**Authors:** Zengzhi Cai, Fan Chen, Ying Jin, Feng Wang

**Affiliations:** ^1^ Department of Medical Oncology Sun Yat‐sen University Cancer Center, State Key Laboratory of Oncology in South China, Collaborative Innovation Center for Cancer Medicine, Sun Yat‐sen University Guangzhou Guangdong China; ^2^ Research Unit of Precision Diagnosis and Treatment for Gastrointestinal Cancer, Chinese Academy of Medical Sciences Guangzhou Guangdong China

**Keywords:** BRAF V600E mutation, circulating tumor DNA, colorectal cancer, HER2 amplification, immunotherapy, KRAS G12C mutation, microsatellite instability, minimal residual disease, precision medicine, targeted therapy

## Abstract

This review summarizes landmark advances in colorectal cancer treatment in 2025, characterizing the transition to dynamic precision oncology. Immunotherapy has revolutionized mismatch repair‐deficient or microsatellite instability‐high patients, becoming the standard first‐line and perioperative regimen. Precision medicine expanded to patients with BRAF V600E, KRAS mutation, and HER2 amplification. While circulating tumor DNA utility was confirmed for surveillance and guiding rechallenge, its role in real‐time treatment adjustment continues to be explored.

## Graphical abstract

Tissue biopsy and molecular profilingenable precise patient stratification into distinct molecular subtypes, which guides the selection of precision treatment. Furthermore, longitudinal liquid biopsies facilitate serial ctDNA monitoring for MRD detection and continuous surveillance. This creates a spatiotemporally dynamic feedback loop, allowing for real‐time treatment adjustments throughout the patient's disease course.

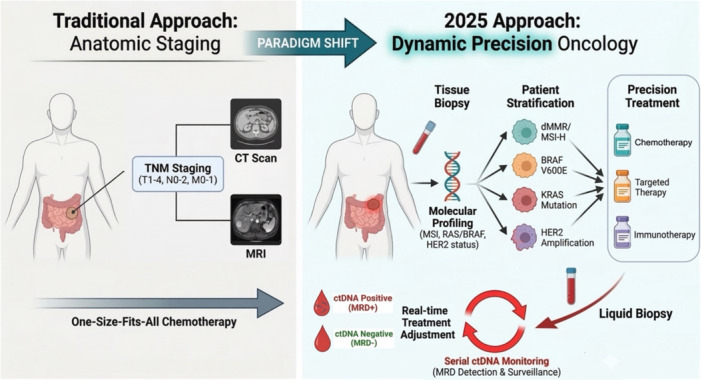

**Abbreviations**
ADCs: antibody‐drug conjugates
CR: complete response
CRC: colorectal cancer
CRT: chemoradiotherapy
ctDNA: circulating tumor DNA
DCR: disease control rate
DFS: disease‐free survival
dMMR: mismatch repair‐deficient
DOR: duration of response
LARC: locally advanced rectal cancer
mCRC: metastatic colorectal cancer
MMR: mismatch repair
mOS: median overall survival
mPFS: median progression‐free survival
MRD: minimal residual disease
MSI: microsatellite instability
MSI‐H: microsatellite instability‐high
NE: not estimable
NR: not reached
ORR: objective response rate
OS: overall survival
pCR: pathological complete response
PFS: progression‐free survival
RFS: recurrence‐free survival
RR: relative risk
SCRT: short‐course radiotherapy
TNT: total neoadjuvant therapy
TRAEs: treatment‐related adverse events

## 1 Introduction: A Paradigm Shift From Anatomic Staging to Dynamic Precision Oncology

The year 2025 marks a pivotal evolution in the systemic management of colorectal cancer (CRC). Driven by a deeper elucidation of tumor molecular biology, the therapeutic landscape has fundamentally shifted from traditional anatomic staging toward a paradigm of precision stratification based on molecular profiling and dynamic management guided by circulating tumor DNA (ctDNA). This year witnessed the comprehensive expansion of immunotherapy for mismatch repair‐deficient (dMMR) or microsatellite instability‐high (MSI‐H) populations—from later‐line settings to the perioperative arena—and the successful targeting of alterations such as BRAF V600E and KRAS G12C. Furthermore, we have seen bold attempts to transition the ctDNA utility from prognostication to interventional guidance. Amidst these global advancements, original contributions from the Chinese oncology community have emerged as critical drivers of disciplinary progress. This review synthesizes key developments in 2025—mostly from the 2025 ASCO annual meeting and ESMO congress—focusing on two core dimensions: molecularly guided precision therapy and ctDNA‐directed whole‐course management. It is important to note that while these conference abstracts represent the cutting edge of oncology, their data are often preliminary; until these findings undergo rigorous peer review and mature follow‐up, their level of evidence remains lower than that of fully published trials and should be interpreted with appropriate clinical caution.

## 2 Precision Therapy Guided by Molecular Subtyping

Therapeutic strategies for CRC are evolving rapidly alongside the clarification of molecular subtypes and the continuous emergence of novel therapeutic modalities. The paradigm has gradually expanded from chemotherapy‐based models to the precision application of targeted therapies and immunotherapies. Treatment for CRC now increasingly emphasizes precision based on mismatch repair (MMR) or microsatellite instability (MSI) status, RAS/BRAF gene status, and HER‐2 status.

### 2.1 dMMR/MSI‐H CRC

Research progress in 2025 indicates that for dMMR/MSI‐H CRC, whether in first‐line metastatic treatment or locally advanced perioperative management, immunotherapy has indisputably become the cornerstone. From the ultra‐long survival data of CheckMate 8HW to the excellent performance of ATOMIC and NeoShot in the perioperative period, all suggest a fundamental shift in treatment strategy for this subtype from *chemotherapy‐dominated to immunotherapy‐prioritized*.

#### 2.1.1 Metastatic CRC (mCRC): Dual Immunotherapy Establishes a New First‐Line Standard

In the first‐line treatment of dMMR/MSI‐H mCRC, dual immunotherapy regimens have demonstrated durable benefits surpassing monotherapy and chemotherapy, redefining the standard of care for this population.

The CheckMate 8HW study is a global, multicenter, phase III trial (*n* = 839) comparing nivolumab plus ipilimumab combination immunotherapy, nivolumab monotherapy, and chemotherapy in the first‐line setting [1]. Preliminary data showed that in the all‐line treatment population, the combination immunotherapy cohort showed a significant progression‐free survival (PFS) advantage over the nivolumab monotherapy cohort (not reached [NR] vs. 39.3 months; hazard ratio [HR] 0.62, *p* = 0.0003). In the first‐line treatment analysis, the median PFS (mPFS) for the combination immunotherapy cohort reached 54.1 months, compared to 5.9 months in the chemotherapy cohort (HR 0.21, *p* < 0.0001) [2]. Grade 3 or 4 treatment‐related adverse events occurred in 78 (22%) and 50 (14%) patients, respectively. There were three treatment‐related deaths: one event of myocarditis and pneumonitis each in the combination immunotherapy cohort and one pneumonitis event in the nivolumab monotherapy cohort. Supported by this robust phase III evidence, dual immunotherapy (nivolumab plus ipilimumab) is strongly recommended as the preferred first‐line standard of care for dMMR/MSI‐H mCRC, superseding both anti‐PD‐1 monotherapy and traditional chemotherapy. Monotherapy may be reserved as an alternative for patients with strict contraindications to anti‐CTLA‐4 agents.

#### 2.1.2 Locally Advanced CRC: A Milestone Breakthrough in Perioperative Immunotherapy

The exploration of immunotherapy in early and locally advanced stages has also achieved remarkable results, gradually replacing traditional chemotherapy as the core treatment in the perioperative setting.

In the adjuvant setting, the ATOMIC phase III trial (*n* = 711) evaluated atezolizumab plus mFOLFOX6 versus chemotherapy alone for the adjuvant treatment of stage III dMMR colon cancer. The results reported at ESMO confirmed that adjuvant immunotherapy combination significantly improved the 3‐year disease‐free survival (DFS), reaching 86.4% versus 76.6% in the chemotherapy cohort (HR 0.50, 95% confidence interval [CI]: 0.35–0.72) [3]. These data provide strong evidence‐based support for adjuvant immunotherapy in stage III dMMR colon cancer, poised to change clinical practice.

In the neoadjuvant setting, the Chinese NeoShot phase II prospective, randomized controlled trial (*n* = 101) systematically evaluated the value of the combination immunotherapy *Shu‐Xin regimen*, which is based on Chinese‐developed drugs. This study compared the IBI310 (an anti‐CTLA‐4 antibody) + sintilimab (an anti‐PD‐1 antibody) versus sintilimab for locally advanced MSI‐H/dMMR colon cancer. Results showed that the pathological complete response (pCR) rate in the combination immunotherapy cohort was as high as 78.4%, significantly superior to 46.7% in the monotherapy cohort (*p* = 0.0015). This pCR rate significantly surpasses historical records for monotherapy (~60%) and some dual‐immunotherapy studies. Notably, 76.2% of enrolled patients belonged to the high‐risk group, yet all patients achieved R0 resection. During a median follow‐up of 21.4 months, no recurrences were observed [4]. The NeoShot study establishes a new landmark in neoadjuvant treatment for MSI‐H/dMMR colon cancer.

Furthermore, comparisons between RESET‐C and NICHE‐2/FOxTROT trials indicate that neoadjuvant immunotherapy, with its extremely high pCR rates, is challenging the traditional surgery‐plus‐chemotherapy model. Results from the RESET‐C phase II study showed that a single cycle of neoadjuvant pembrolizumab could induce high rates of pathological response in dMMR stage I–III patients (pCR rate 61% for stage I–II, 33% for stage III) [5]. A pooled analysis of NICHE‐2 (neoadjuvant dual‐immunotherapy) and FOxTROT (neoadjuvant chemotherapy) at ESMO revealed that neoadjuvant immunotherapy was significantly superior to chemotherapy in terms of survival benefit. The 3‐year DFS rate in NICHE‐2 reached 100%, whereas it was only 80% for patients receiving neoadjuvant chemotherapy (FOxTROT study) [6].

Collectively, in the adjuvant setting, the addition of atezolizumab to standard chemotherapy is highly recommended for stage III dMMR colon cancer. In the neoadjuvant setting, despite the data primarily originating from phase II trials, the unprecedented pCR rates position neoadjuvant immunotherapy (with or without CTLA‐4 blockade) as a highly prioritized, paradigm‐shifting option for locally advanced dMMR/MSI‐H tumors, particularly for patients desiring organ preservation or facing high surgical morbidity.

### 2.2 BRAF V600E Mutant CRC: First‐Line Targeted Combination Rewrites Prognosis With Significant Survival Benefit

BRAF V600E mutations account for approximately 8%–15% of mCRC and are traditionally characterized by high aggressiveness, poor chemosensitivity, and extremely poor prognosis [7, 8]. In 2025, with the full disclosure of data from the BREAKWATER study, the treatment landscape for this field has been fundamentally reversed, transforming from a refractory subtype to a model of precision therapy offering extended survival.

BREAKWATER, an open‐label, phase III trial (*n* = 236), evaluated the BRAF inhibitor encorafenib combined with the anti‐EGFR antibody cetuximab and chemotherapy (mFOLFOX6) (the EC+Chemo cohort) versus current standard of care (investigator's choice of chemotherapy combined with anti‐angiogenic or anti‐EGFR agents) in the first‐line treatment. The study showed that mPFS reached 12.8 months, significantly superior to 7.1 months in the control cohort (HR 0.53, 95% CI: 0.44–0.65, *p* < 0.0001), reducing the risk of disease progression or death by 47%. Importantly, the median overall survival (mOS) for the EC + Chemo cohort broke through to 30.3 months versus 15.1 months in the control cohort (HR 0.49, 95% CI: 0.39–0.61, *p* < 0.001) [9].

Interestingly, according to results presented at ESMO, analysis for Chinese population (*n* = 78) showed that for EC+Chemo cohort versus control cohort, mPFS was 10.0 months versus 7.1 months (HR 0.63, 95% CI: 0.33–1.21), and mOS was not estimable (NE) versus 13.4 months (HR 0.34, 95% CI: 0.18–0.66), correspondingly [10]. This indicates that racial differences did not diminish the efficacy of this combination regimen, supporting its broad application in Chinese patients with BRAF V600E mutations.

Based on the definitive phase III BREAKWATER trial, the targeted triplet regimen (encorafenib + cetuximab + mFOLFOX6) should be rapidly adopted as the new standard‐of‐care for the first‐line treatment of BRAF V600E‐mutated mCRC. Traditional chemotherapy combined with bevacizumab should be relegated to a secondary option for this specific molecular subtype.

### 2.3 KRAS Mutant CRC: Combination Therapy Overcomes the Undruggable Challenge and G12C Targeting Sees Comprehensive Expansion

KRAS mutations have long been regarded as a significant therapeutic challenge in CRC, accounting for 40%–50% of mCRC patients [7, 8]. In 2025, with the maturation of combination treatment strategies for KRAS G12C mutations and the emergence of next‐generation inhibitors and domestic original drugs, the treatment landscape is undergoing a historic transition from *undruggable to precision combination*.

#### 2.3.1 KRAS G12C Inhibitors: Combination With Anti‐EGFR Therapy Establishes Core Status

Previous studies indicated that CRC has a significant feedback activation mechanism of the EGFR signaling pathway, limiting the efficacy of KRAS G12C inhibitor monotherapy [11]. Multiple studies published in 2025 further solidified the combination strategy of *KRAS inhibitor + anti‐EGFR antibody ± chemotherapy*.

CodeBreaK 300, a phase III trial (*n* = 160), explored the efficacy of sotorasib (a KRAS G12C inhibitor, 960 mg) with panitumumab (an anti‐EGFR antibody) after failure of standard treatment. The result showed that for the combination cohort versus standard third‐line treatment (regorafenib or TAS‐102), mPFS was 5.7 versus 2.0 months (HR 0.45, 95% CI: 0.29–0.72), and mOS was NE versus 10.3 months (HR 0.70, 95% CI: 0.41–1.18, *p* = 0.20) [12]. Furthermore, The CodeBreaK 101, a phase 1b trial (*n* = 40), explored the sotorasib + panitumumab + chemotherapy (FOLFIRI) in pre‐treated KRAS G12C‐mutated mCRC. After a median follow‐up of 29.2 months, the triplet regimen cohort achieved an objective response rate (ORR) as high as 57.5% (95% CI: 40.9%–73.0%), superior to historical data for KRAS inhibitor monotherapy (~20%) [13]. Updated survival data presented at the ASCO demonstrated that the mPFS reached 8.2 months (95% CI: 7.0–10.8), with mOS 17.9 months (95% CI: 12.9–25.1) [14].

NCT04585035, an open‐label, nonrandomized phase II trial (*n* = 68), assessed the safety and efficacy of garsorasib ± cetuximab in KRAS G12C‐mutated CRC. In the monotherapy cohort (*n* = 26), ORR was 19.2% (95% CI: 6.6–39.4), disease control rate (DCR) was 92.3% (95% CI: 74.9–99.1), median PFS was 5.5 months (95% CI: 2.9–11.6), and mOS was 13.1 months (95% CI: 9.5–NE). In the combination cohort (*n* = 42), ORR was 45.2% (95% CI: 29.8–61.3), DCR was 92.9% (95% CI: 80.5–98.5), mPFS was 7.5 months (95% CI: 5.5–8.1), and mOS was not reached [15].

These three results strongly validated that *dual‐target blockade* (simultaneous inhibition of KRAS and EGFR) with chemotherapy backbone can partially overcome resistance, which is strongly recommended as the standard‐of‐care in the third‐line setting or beyond.

#### 2.3.2 Next‐Generation Inhibitors: Olomorasib, MK‐1084, and Fulzerasib Demonstrate Strong Potential

In addition to Sotorasib, next‐generation KRAS G12C inhibitors are attempting to further improve efficacy and safety through optimized molecular structures and binding characteristics.

NCT04956640, a phase 1/2 trial (*n* = 93), explored the efficacy of olomorasib, a highly selective and potent next‐generation KRAS G12C inhibitor, with cetuximab in patients with first‐line treatment failure. According to preliminary survival outcomes reported at ASCO, the combination cohort reached an mPFS of 7.5 months (95% CI: 6.6–8.8), with an ORR of 42% [16].

KANDLELIT‐001, a phase 1/2 trial (*n* = 153), explored the efficacy of MK‐1084, a next‐generation KRAS G12C‐GDP covalent inhibitor, with or without cetuximab and/or chemotherapy. The results presented at ASCO showed that ORR was 50% in the MK‐1084 plus cetuximab cohort and 14% in the MK‐1084 + cetuximab + mFOLFOX6 cohort [17], both of which showed potential efficacy. The PFS and OS data are not mature.

Data pooled from two phase I clinical studies (NCT05005234 and NCT05497336, *n* = 56 totally) showed that fulzerasib, a China‐developed KRAS G12C inhibitor, showed an ORR of 44.6% (95% CI: 31.3%–58.5%), an mPFS of 8.1 months (95% CI: 5.5–13.8), and an mOS of 17.0 months (95% CI: 12.6–NE) for mCRC [18]. These monotherapy data approach or even partially surpass the combination scheme data of international peers, indicating that Chinese scholars have also made breakthrough progress in the exploration of the KRAS G12C field.

### 2.4 HER‐2 Positive CRC: ADC Drugs Solidify Later‐Line Standards and First‐Line Prognostic Value Further Established

HER‐2 amplification or overexpression accounts for approximately 2%–5% of mCRC, with a higher proportion in RAS/BRAF wild‐type patients [19]. In 2025, with the maturation of data on novel antibody‐drug conjugates (ADCs), dual Her2 blockade regimen, and the publication of large‐scale prognostic analyses, the precision diagnostic and therapeutic pathway for this subtype has become clearer.

#### 2.4.1 Later‐Line Treatment: T‐DXd Sets New Benchmark, Breaking RAS Mutation Limitations

In the later‐line treatment of HER‐2 positive mCRC, the position of the novel ADC drug fam‐trastuzumab deruxtecan (T‐DXd) and dual HER2 blockade therapy has been further consolidated.

DESTINY‐CRC02, a phase II trial (*n* = 122), which explored the efficacy of T‐DXd in HER‐2 positive mCRC with first‐line treatment failure, confirmed that 5.4 mg/kg is the optimal recommended dose for T‐DXd. Updated survival data presented at the ESMO showed that in previously treated HER‐2 positive mCRC patients, the 5.4 mg/kg dose cohort achieved an ORR of 37.8% (95% CI: 27.3–49.2), an mOS of 15.9 months (95% CI: 12.6–18.8), and a median duration of response (DOR) of 5.5 months (95% CI: 4.2–8.1). The study showed that T‐DXd was equally effective in patients with RAS mutations co‐occurring with HER‐2 amplification [20]. This finding is clinically significant, suggesting that HER‐2 amplification may be a more dominant driver than RAS mutations, and T‐DXd is expected to be an effective rescue method for such patients previously considered refractory.

#### 2.4.2 Chemotherapy‐Free Regimens: Dual HER2 Blockade With Tucatinib Demonstrates Sustained Clinical Benefit

MOUNTAINEER, a multicenter, open‐label, phase II trial (*n* = 84), has explored the clinical utility of a chemotherapy‐free regimen, which consists of tucatinib (an oral, highly selective HER2 tyrosine kinase inhibitor) and trastuzumab (a HER2‐targeted monoclonal antibody), in chemotherapy‐refractory HER‐2 positive mCRC. With a mature median follow‐up of 32.4 months, the study reported an ORR of 39.3% and a notably durable median DOR of 15.2 months [21]. This study provides evidence for the dual HER2‐targeted, chemotherapy‐free combination of tucatinib plus trastuzumab to be an effective regimen in the later‐line treatment of mCRC.

#### 2.4.3 Prognostic and Predictive Value: HER‐2 Is a Negative Factor in the RAS/BRAF Wild‐Type Population

A study pooled data from RAS/BRAF wild‐type mCRC patients across eight randomized controlled trials (*n* = 1604), and preliminary results reported at ASCO showed that HER‐2 amplification or overexpression is an independent negative prognostic factor. Compared with HER‐2 negative patients, HER‐2 positive patients (*n* = 81) had significantly shorter mPFS (9.8 vs. 12.2 months, HR 1.31, *p* = 0.02) and mOS (28.0 vs. 34.9 months, HR 1.37, *p* = 0.01). Furthermore, analysis of anti‐EGFR efficacy found that HER‐2 positive patients had lower sensitivity to anti‐EGFR antibodies (e.g., cetuximab, panitumumab), with efficacy comparable to bevacizumab, failing to demonstrate the advantage anti‐EGFR drugs typically offer in the RAS/BRAF wild‐type population [22]. This strongly supports mandatory HER‐2 testing when diagnosing RAS/BRAF wild‐type mCRC and cautions against blind first‐line use of anti‐EGFR antibodies in HER‐2 positive patients, prompting a critical re‐evaluation of whether anti‐HER2 targeted therapies should be *moved to earlier lines* of treatment rather than being reserved for later stages.

### 2.5 Target‐Negative pMMR/MSS Subtypes

#### 2.5.1 Treatment Strategy of Anti‐EGFR Antibody With Chemotherapy in the First‐Line Therapy of RAS Wild‐type mCRC: De‐escalation and Optimization

Research in 2025 shifted focus from treatment intensification to the value of de‐escalation and intermittent strategies.

TRIPLETE, a phase III trial (*n* = 435), compared intensified chemotherapy (mFOLFOXIRI, triplet) plus panitumumab versus standard chemotherapy (mFOLFOX, doublet) plus panitumumab in RAS/BRAF wild‐type mCRC patients. Results showed that mOS for the intensified cohort was inferior to the control cohort (33.3 vs. 41.1 months; HR 0.79, 95% CI: 0.63–0.99, *p* = 0.049) [23]. The OS results showed that the intensified cohort not only failed to demonstrate a survival benefit but also limited subsequent treatments due to greater toxicity.

IMPROVE, an open‐label phase II trial (*n* = 69), explored an intermittent strategy—induction treatment followed by a pause in panitumumab after disease control, reintroducing it upon progression. Results showed that the intermittent cohort significantly prolonged PFS on treatment than the control cohort (17.5 vs. 11.2 months) while significantly reducing skin toxicity (17.9% vs. 30.3% in overall rate of grade > 2 skin‐related adverse effects), suggesting it as a safe and effective strategy for delaying resistance [24].

CAPCET, an open‐label phase II trial (*n* = 168), confirmed that mPFS of mCapOx (capecitabine 1000 mg/m^2^ orally twice daily on Day 1–7, and oxaliplatin 85 mg/m^2^ iv. on Day 1) plus cetuximab was comparable to traditional mFOLFOX6 plus cetuximab (12.7 vs. 12.0 months), according to preliminary data presented at ASCO [25]. The new regimen significantly improves convenience by being pump‐free.

Based on the current evidence, standard doublet chemotherapy combined with an anti‐EGFR antibody remains the preferred first‐line priority for left‐sided RAS/BRAF wild‐type mCRC. Furthermore, the mCapOx plus cetuximab regimen might be considered a prioritized alternative for patients preferring outpatient management to avoid the inconvenience of infusion pumps, and phase III trial is warranted.

#### 2.5.2 Oral VEGFR‐TKI in the First‐Line Therapy for RAS/BRAF Wild‐Type mCRC

ANCHOR, a multicenter, prospective, randomized, phase III trial (*n* = 748), compares the efficacy of anlotinib (an oral VEGFR‐TKI) versus bevacizumab (an anti‐VEGF antibody) with standard first‐line chemotherapy among patients with RAS/BRAF wild‐type mCRC. As reported at the ASCO, mPFS in the anlotinib and bevacizumab cohort was comparable (11.04 vs. 11.04 months, HR 1.00, 95% CI: 0.84–1.18). Serious treatment‐related adverse events (TRAEs) occurred in 143 (38.34%) of 373 patients in the anlotinib cohort and in 129 of 375 (34.40%) patients in the bevacizumab cohort [26]. The results provide a new treatment option for unresectable RAS/BRAF wild‐type mCRC patients. While intravenous bevacizumab or anti‐EGFR antibodies combined with doublet chemotherapy remain the conventional standard first‐line regimens, the ANCHOR study elevates oral VEGFR‐TKIs to a viable alternative. Anlotinib is particularly recommended as a first‐line option for patients who strongly favor oral administration convenience, those with poor venous access aiming to avoid central venous catheters, or patients requiring a more flexible outpatient treatment schedule, offering non‐inferior efficacy to standard intravenous anti‐angiogenic therapy.

#### 2.5.3 Immunotherapy in the Third Line Therapy for pMMR/MSS mCRC

STELLAR‐303, a randomized open‐label phase III trial (*n* = 901), compared the efficacy of zanzalintinib (a novel inhibitor of multiple receptor tyrosine kinases) + atezolizumab (an anti‐PD‐L1 antibody) versus regorafenib (an inhibitor of multiple receptor tyrosine kinases). Zanzalintinib + atezolizumab cohort significantly improved mOS (10.9 vs. 9.4 months; HR 0.80, 95% CI: 0.69–0.93, *p* = 0.0045) and mPFS (3.7 vs. 2.0 months, HR 0.68, 95% CI: 0.59–0.79) versus regorafenib cohort. Grade 3/4 TRAEs occurred in 59% combination cohort and 37% monotherapy cohort [27]. The results indicated that immunotherapy with target therapy could provide survival benefits for patients with second‐line therapy failure.

### 2.6 Integration of Systemic Therapy With Surgery and Radiotherapy

#### 2.6.1 Radiotherapy and Immunotherapy in Locally Advanced Rectal Cancer (LARC)

LARC is characterized by tumors classified as T3 or T4, or the presence of node‐positive disease, in the absence of metastatic spread [28]. Current ASCO guidelines advocate for total neoadjuvant therapy (TNT), integrating a CAPOX or FOLFOX chemotherapy backbone with chemoradiotherapy (CRT), followed by total mesorectal excision surgery or watchful waiting, for patients presenting with pMMR/MSS LARC [29]. Despite this multimodal approach, a recent meta‐analysis demonstrates that the pathologic complete response (pCR) rate remains modest, at approximately 20.9% [30]. This limitation has prompted investigations into the efficacy of incorporating PD‐1 inhibitors into neoadjuvant chemotherapy regimens.

POLARSTAR, a randomized phase II trial (*n* = 186), explored the efficacy and safety of the radiation–immune checkpoint inhibitor combination. Patients from eight centers were randomly assigned to receive neoadjuvant chemoradiation + concurrent/sequential PD‐1 blockade or neoadjuvant chemoradiation alone. The results showed that the pCR rate in the CRT plus sequential anti‐PD‐1 cohort was significantly higher than that in the CRT alone cohort (29.5% vs. 12.9%; RR 2.287, 95% CI: 1.076–4.861, *p* = 0.024), Interestingly, concurrent administration *did not* yield a statistically significant improvement (25.4% vs. 12.9%; relative risk [RR] 1.968, 95% CI: 0.909–4.263, *p* = 0.076). Importantly, the addition of PD‐1 blockade raised no substantial safety concerns, as no significant differences were observed between the experimental and control cohorts regarding adverse reactions, surgical complications, or disease progression [31]. The results show that adding PD‐1 blockade after neoadjuvant chemoradiation increases the pCR rate for patients with LARC and raises no substantial safety concerns.

SPRING‐01, a randomized, single‐center, open‐label, phase II trial (*n* = 98), assessed the addition of the PD‐1 inhibitor sintilimab to short‐course radiotherapy (SCRT) followed by CAPOX. The cohort receiving sintilimab plus CAPOX achieved a significantly higher pCR rate than the CAPOX‐alone cohort (29% vs. 16%, *p* = 0.015). Furthermore, the integration of immunotherapy did not substantially increase overall toxicity; the incidence of grade 3–4 adverse events was comparable between the two cohorts (33% vs. 35%) [32].

UNION TNT, a multicenter, single‐arm, open‐label, phase II trial (*n* = 45), explored the efficacy and safety of adding the VEGFR inhibitor fruquintinib and the PD‐L1 inhibitor adebrelimab to SCRT followed by CAPOX as a TNT for patients with high‐risk LARC. The results showed that the treatment strategy provided an impressive overall complete response (CR) rate of 65% among evaluable patients. Specifically, for the cohort that proceeded to surgery, the pCR rate was 63.16%, alongside a 100% R0 resection rate. Regarding safety, grade 3–4 adverse events occurred in 47.1% of patients. Notably, four serious adverse events of intestinal perforation were reported, although all these patients subsequently underwent surgical intervention and achieved a pCR [33], which indicates that adding targeted anti‐angiogenic therapy and PD‐L1 blockade to neoadjuvant SCRT and chemotherapy yields a highly favorable pCR rate for high‐risk LARC, while the safety profile—particularly regarding severe gastrointestinal toxicity—requires careful management and monitoring.

Collectively, these findings strongly support the integration of PD‐1 blockade into standard neoadjuvant for LARC and warrant further validation through large‐scale phase III trials. Building on this promising foundation, optimizing these regimens by incorporating targeted therapies, such as anti‐angiogenic agents, represents a critical next frontier to further maximize immunotherapeutic outcomes.

#### 2.6.2 Systemic Therapy Combined With Surgery or Local Ablation for mCRC

The efficacy of systemic medical treatment is fundamentally tied to its capacity to facilitate curative‐intent local interventions for metastatic disease. In 2025, several critical trials further delineated the optimal integration of systemic regimens with surgical and local ablative strategies, particularly for colorectal liver metastases.

COLLISION, a randomized phase III trial (*n* = 300), explored the efficacy and safety of thermal ablation versus standard surgical resection in patients with limited (≤ 10 lesions) and small‐sized (≤ 3 cm) colorectal liver metastases. With a median follow‐up of 28.9 months, the trial was stopped early after meeting prespecified criteria for non‐inferiority and superior safety. The results showed that OS in the thermal ablation cohort was non‐inferior to the surgical resection cohort (mOS not reached in both cohorts; HR 1.05, 95% CI: 0.69–1.58, *p* = 0.83). Additionally, local control was comparable between the two treatment strategies (HR 0.13, 95% CI: 0.02–1.06, *p* = 0.057). Regarding safety, both overall adverse events (19% vs. 46%, *p* < 0.0001) and serious adverse events (7% vs. 20%) were markedly lower in the thermal ablation cohort, and 3 treatment‐related deaths (2%) occurred in the surgical resection cohort [34]. The results show that thermal ablation yields non‐inferior survival outcomes and a significantly better safety profile compared to surgical resection, indicating that it should be considered a preferred, individualized treatment option for patients with small‐volume colorectal liver metastases.

TransMet, an open‐label, randomized phase trial (*n* = 94), explored the efficacy and safety of liver transplantation combined with chemotherapy versus chemotherapy alone. Patients with permanently unresectable, liver‐only metastases from BRAF‐non‐mutated CRC responsive to systemic chemotherapy were randomly assigned to receive liver transplantation + chemotherapy or to continue chemotherapy alone. The results showed that the 5‐year OS rate in the liver transplantation + chemotherapy cohort was significantly higher than the chemotherapy alone cohort (56.6% vs. 12.6%; HR 0.37, 95% CI: 0.21–0.65, *p* = 0.0003). Importantly, the integration of liver transplantation raised no substantial excess safety concerns compared to standard care, though one postoperative death due to multi‐organ failure was reported in the transplant cohort [35]. The results show that combining liver transplantation with chemotherapy significantly improves long‐term survival for highly selected patients with permanently unresectable colorectal liver metastases, indicating it as a new standard therapeutic option.

Collectively, the integration of systemic chemotherapy with tailored local interventions optimizes long‐term survival while maintaining acceptable safety profiles. Moving forward, the precise role and extent of local consolidation in oligometastatic settings warrant further prospective validation. The ongoing phase III Alliance A022101/NRG‐GI009 trial, which evaluates total ablative therapy combined with standard systemic regimens [36], is anticipated to provide definitive evidence for establishing a standard multimodal curative paradigm in limited mCRC.

## 3 Exploration of ctDNA and Minimal Residual Disease (MRD) Guided Therapy

With the maturation of liquid biopsy technology, ctDNA has evolved from a research tool into a pivotal biomarker for whole‐course CRC management. Two primary methodologies have been developed for ctDNA‐based MRD detection: tumor‐informed strategies, which identify tumor‐specific mutations through initial tissue sequencing to guide ctDNA monitoring, and tumor‐agnostic approaches, which utilize predefined panels to detect common cancer‐associated genomic or epigenomic alterations directly from plasma without prior tissue analysis [37]. In 2025, the release of multiple landmark studies further solidified ctDNA as a robust prognostic factor, while simultaneously revealing the complexities of guiding clinical intervention—namely, while prognostic stratification is established, predictive utility for treatment modification remains a challenge.

### 3.1 Adjuvant Treatment Stage: The Dilemma of Intervention Strategies

In the postoperative adjuvant setting for early‐to‐mid‐stage CRC, tailoring treatment intensity based on ctDNA status is a major area of investigation. However, several key Phase III studies published in 2025 suggest that simply adjusting chemotherapy intensity based on MRD positivity has not yet translated into definitive survival benefits.

DYNAMIC‐III, a multi‐center randomized phase II/III trial (*n* = 961), challenged the limits of escalating treatment intensity based on ctDNA for stage III colon cancer patients. For postoperative ctDNA‐positive high‐risk patients, it attempted to upgrade adjuvant chemotherapy from standard FOLFOX to FOLFOXIRI (triplet chemotherapy). Unfortunately, the study presented at ASCO did not meet its primary endpoint. In 259 ctDNA‐positive patients, even with intensified treatment (FOLFOXIRI), the 2‐year recurrence‐free survival (RFS) was 52% (*n* = 129), with no significant difference compared to the standard treatment cohort (61%, *n* = 130) (HR 1.11, 90% CI: 0.83–1.48, *p* = 0.6) [38]. In 702 ctDNA‐negative patients, de‐escalation reduced oxaliplatin use (34.8% vs. 88.6%, RR 0.41, *p* < 0.001) and hospitalizations (8.5% vs. 13.2%, RR 0.64, *p* = 0.047), but yielded slightly lower RFS than standard management (85.3% vs. 88.1%, absolute difference −2.8%, 95% CI: −8%–2.3%), not meeting the non‐inferiority margin, failing to confirm non‐inferiority. In comparison, however, for the T_1‐3_N_1_ low‐risk subgroup, de‐escalated treatment had a 3‐year RFS comparable to the standard cohort (91.0% vs. 93.2%, absolute difference −2.2%, 95% CI: −7.2% to 2.7%) [39].

PEGASUS, a multicenter, investigator‐initiated trial (*n* = 135), explored adjuvant benefits guided by ctDNA in high‐risk stage II/stage III CRC. In this study, 100 ctDNA‐negative patients received 6 months of Capecitabine monotherapy, while 35 ctDNA‐positive patients received 3 months of CAPOX combination therapy. According to the results presented at ESMO, after a median follow‐up of 33.5 months, 12 recurrences occurred in ctDNA‐negative patients (2‐year disease‐free survival rate 88%, missing the prespecified threshold of 92%). Among 35 ctDNA‐positive patients, 13 (37%) recurrences were observed. Although the primary endpoint for superiority was not met, the recurrence risk observed in ctDNA‐positive patients was much higher than in ctDNA‐negative patients (2‐year time‐to‐relapse rate: 61.5% vs. 87.6%, HR 3.22, 95% CI: 1.20–7.96, *p* = 0.0013), and patients who converted to negative through treatment had a significantly improved prognosis [40].

ALTAIR, a double‐blind phase III trial, screened patients from the GALAXY study who were persistently ctDNA‐positive post‐surgery but radiologically recurrence‐free (*n* = 243), where patients were randomized to TAS‐102 or placebo. Results showed that TAS‐102 failed to significantly improve DFS in the overall population (median DFS: 9.30 vs. 5.55 months, HR 0.79, 95% CI: 0.60–1.05, *p* = 0.107), according to data presented at ESMO. However, significant benefit was shown in the subgroup of resected Stage IV/M_1a_ patients (*n* = 66, HR 0.53, *p* = 0.012), suggesting differential sensitivity to MRD intervention across disease stages [41].

The failure of landmark trials such as DYNAMIC‐III and PEGASUS to achieve their primary superiority endpoints necessitates a critical re‐evaluation of MRD‐guided interventional strategies. First, these results highlight that while ctDNA positivity indicates high biological aggressiveness, mere intensification of conventional chemotherapy may have reached a ceiling of efficacy, necessitating interventions such as targeted therapy or immunotherapy. More importantly, a certain group of patients with postoperative ctDNA positivity may reflect inherently chemoresistant micrometastatic clones; therefore, overcoming this biological hurdle may require a paradigm shift from cytotoxic escalation toward the integration of novel mechanisms. Second, the recurrent events observed in the ctDNA‐negative arms of these trials highlight the critical limitation of current assay sensitivity. Relying on a single threshold for ctDNA negativity risks generating false negatives, particularly in non‐shedding tumors or extremely low‐burden disease, making blunt treatment de‐escalation hazardous. Conversely, the safety of de‐escalation in ctDNA‐negative patients requires careful patient selection, particularly within low‐risk subgroups. Third, relying on a single, static postoperative snapshot may miss the optimal therapeutic window. If available, future trial designs may incorporate longitudinal ctDNA clearance kinetics, such as evaluating dynamic changes during and immediately after early intervention, rather than depending on binary baseline results.

### 3.2 Monitoring and Early Intervention

Although chemotherapy intervention studies yielded mixed results, the value of ctDNA in monitoring recurrence and guiding surgical decisions was strongly validated.

FIND, a prospective multicenter randomized phase III trial (*n* = 728), included post‐operative non‐metastatic CRC patients, comparing ctDNA methylation monitoring with traditional standard monitoring. Results presented at ESMO showed that the ctDNA‐guided monitoring strategy significantly increased the proportion of recurrent patients receiving curative resection of metastases (50% vs. 22.6%, RR 2.214, 95% CI: 1.060–4.780, *p* = 0.034), directly translating into potential survival benefits [42].

INTERCEPT, a prospective study (*n* = 1301), analyzed dynamic changes in ctDNA‐positive stage I‐IV CRC patients (*n* = 402). As reported at the ASCO, the study found that adjuvant therapy could convert approximately 26% (20/77) of post‐operative ctDNA‐positive patients to negative, and this ctDNA clearance was strongly correlated with superior long‐term survival. In contrast, spontaneous ctDNA clearance was rare (4%), persistent ctDNA clearance was more rare (1.7%), establishing *ctDNA clearance* as a core early efficacy endpoint for adjuvant therapy [43].

### 3.3 Precision Guidance for Later‐Line Targeted Therapy Rechallenge

In advanced CRC, ctDNA has become a crucial tool guiding treatment decision, especially regarding the reintroduction of anti‐EGFR therapies.

CITRIC, a multicenter randomized phase II clinical trial (*n* = 114), confirmed that after resistance to first‐line anti‐EGFR therapy, rechallenge with cetuximab + irinotecan improved outcomes, but only in patients whose ctDNA testing confirmed RAS/BRAF wild‐type status. This strategy improved disease control rate (24% vs. 12%, *p* = 0.01), mPFS (4.8 vs. 2.7 months, HR 0.6, 95% CI: 0.35–1.03, *p* = 0.06), and mOS (11.3 vs. 7.4 months, HR 0.8, 95% CI: 0.44–1.57, *p* = 0.6) [44], according to preliminary data presented at ESMO.

PARERE, an open‐label, multicenter, randomized phase II trial (*n* = 213), compared panitumumab versus regorafenib in mCRC patients with resistance to first‐line anti‐EGFR therapy, specifically in those confirmed as RAS/BRAF wild‐type by ctDNA. Results presented at ESMO showed that panitumumab was superior to regorafenib in ORR (ORR1: 16% vs. 2%, *p* < 0.01; ORR2: 17% vs. 0%, *p* < 0.01), DCR (DCR1: 59% vs. 32%, *p* < 0.01; DCR2: 56% vs. 37%, *p* = 0.02), and PFS (PFS1: 4.2 vs. 2.3 months, *p* = 0.128; PFS2: 3.9 vs. 2.7 months, *p* = 0.026) [45].

These findings underscore that blind re‐challenge with anti‐EGFR therapy offers limited benefit. Liquid biopsy serves as an effective tool for negative selection. Only when ctDNA testing confirms the complete disappearance of resistance mutations (RAS/BRAF/EGFR extracellular domain) and the tumor reverts to a wild‐type state can the rechallenge strategy truly translate into survival benefits.

### 3.4 Navigating the Challenges of Spatiotemporal Dynamic Management

The primary goal of MRD assessment is to achieve precise patient stratification. While identifying MRD‐positive patients has become technically feasible, the core challenge now lies in choosing the right escalation strategy. It remains to be explored whether specific mutational profiles or varying ctDNA levels require personalized intensification, such as targeted therapy or immunotherapy, rather than a standard increase in chemotherapy.

For MRD‐negative patients, the main bottleneck is no longer just technology, but tumor biology. Beyond the need for deep sequencing and sophisticated bioinformatic algorithms [46, 47], the non‐shedding phenotype remains a major hurdle. Dormant micrometastases may release so little DNA that they fall below the detection limit. Therefore, *test negativity does not necessarily imply biological negativity or biological cure*. To truly define low‐risk, it is important to look beyond genomics and incorporate clinical history, imaging, and pathology. Specifically, pathology AI can decode the tumor microenvironment from surgical slides, identifying high‐risk features like vascular invasion that ctDNA might miss [48], which may identify high‐risk ctDNA‐negative patients [49].

More importantly, risk is not static. Since tumors and their microenvironments evolve under treatment pressure, a single snapshot assessment is insufficient. The field is moving toward longitudinal surveillance, where repeated testing captures the dynamic nature of the disease. This shift from a one‐time decision to a continuous intervention model requires a fundamental change in how we design clinical trials. Traditional, rigid trials are no longer adequate. Instead, we need adaptive master protocols, such as umbrella or platform trials, that allow for real‐time therapy adjustments as soon as a molecular recurrence is detected.

## 4 Unique Contributions of Chinese Clinical Research to Global CRC Management

While the detailed efficacy outcomes of individual trials have been discussed in their respective molecular sub‐sections above, a macroscopic analysis reveals that Chinese clinical research in 2025 systematically contributed to the global CRC landscape through following distinct strategic dimensions.

### 4.1 Transitioning From *Fast‐Follower to Co‐Leader* in Drug Development

In the realm of novel targets and immunotherapies, Chinese domestic innovations have demonstrated global competitiveness. The independent development of domestic KRAS G12C inhibitors (e.g., fulzerasib) and novel immune‐combinations (such as the Shu‐Xin regimen in the NeoShot trial) illustrates a paradigm shift. Rather than relying on imported agents, these Chinese proprietary drugs achieved profound pathological responses that parallel, and even challenge, the benchmarks set by international combination regimens. This rapid indigenous development not only diversifies the global therapeutic arsenal but also plays a vital role in mitigating drug accessibility and economic burdens in developing nations.

### 4.2 Strategic Focus on Clinical Accessibility and Outpatient Optimization

Another characteristic of Chinese clinical trials is their strong emphasis on adapting cutting‐edge treatments to high‐volume clinical realities. While international studies often focus strictly on escalating efficacy, Chinese research uniquely championed de‐escalation and convenience. Trials such as CAPCET and ANCHOR strategically utilized oral targeted agents and pump‐free systemic regimens. These innovations directly address the practical bottleneck of hospital bed shortages, significantly reducing hospitalization dependency and improving patient quality of life without sacrificing oncological outcomes.

## 5 Conclusion

### 5.1 2025 in Retrospect: The Maturation of Precision and Immunotherapy

The year 2025 represents a landmark period of harvest and transformation in the precision treatment of CRC. We have witnessed the dMMR/MSI‐H population officially entering the era of curative immunotherapy; formerly intractable targets like BRAF V600E and KRAS G12C have been successfully targeted; and diagnostic and therapeutic pathways for HER‐2 amplification and RAS wild‐type patients have achieved unprecedented refinement. Notably, high‐quality original research from China, exemplified by the Neoshot and CAPCET studies and the development of domestic KRAS inhibitors, has marked a transition from emulation to leadership, contributing pivotal evidence to the global CRC landscape.

### 5.2 Future Perspectives: Navigating the Challenges of Spatiotemporal Dynamic Management

Looking forward, the treatment paradigm for CRC is accelerating from static classification to dynamic precision management. The era of relying on ctDNA to adjust chemotherapy intensity is drawing to a close. The future direction lies in the deep integration of molecular subtyping and dynamic ctDNA monitoring. We must move beyond merely *detecting* recurrence via ctDNA to leveraging real‐time molecular profiling for the precise intervention with immunotherapy or potent novel targeted agents. This spatiotemporally dynamic and individualized management strategy promises to drive the transition from monitoring recurrence to curing recurrence, ultimately securing optimal survival outcomes for every patient.

## Author Contributions

**Zengzhi Cai:** writing – original draft (lead), writing – review and editing (equal). **Fan Chen:** supervision (equal), writing – review and editing (equal). **Ying Jin:** conceptualization (lead), supervision (equal), writing – review and editing (equal). **Feng Wang:** funding acquisition (lead), supervision (lead), writing – review and editing (lead).

## Ethics Statement

The authors have nothing to report.

## Consent

The authors have nothing to report.

## Conflicts of Interest

The authors declare no conflicts of interest.

## Data Availability

Data sharing is not applicable to this article as no data sets were generated or analyzed during the current study.
